# Optimal back-extrapolation method for estimating plasma volume in humans using the indocyanine green dilution method

**DOI:** 10.1186/1742-4682-11-33

**Published:** 2014-07-22

**Authors:** David Polidori, Clarence Rowley

**Affiliations:** 1 Cardiovascular and Metabolism, Janssen Research & Development, LLC, 3210 Merryfield Row, San Diego, CA 92121, USA; 2 Mechanical and Aerospace Engineering Department, D-wing, Engineering Quadrangle, Princeton University, Princeton, NJ 08544, USA

**Keywords:** Clinical pharmacology, Clinical research, Pharmacokinetics and drug metabolism, Pharmacology, Physiology

## Abstract

**Background:**

The indocyanine green dilution method is one of the methods available to estimate plasma volume, although some researchers have questioned the accuracy of this method.

**Methods:**

We developed a new, physiologically based mathematical model of indocyanine green kinetics that more accurately represents indocyanine green kinetics during the first few minutes postinjection than what is assumed when using the traditional mono-exponential back-extrapolation method. The mathematical model is used to develop an optimal back-extrapolation method for estimating plasma volume based on simulated indocyanine green kinetics obtained from the physiological model.

**Results:**

Results from a clinical study using the indocyanine green dilution method in 36 subjects with type 2 diabetes indicate that the estimated plasma volumes are considerably lower when using the traditional back-extrapolation method than when using the proposed back-extrapolation method (mean (standard deviation) plasma volume = 26.8 (5.4) mL/kg for the traditional method vs 35.1 (7.0) mL/kg for the proposed method). The results obtained using the proposed method are more consistent with previously reported plasma volume values.

**Conclusions:**

Based on the more physiological representation of indocyanine green kinetics and greater consistency with previously reported plasma volume values, the new back-extrapolation method is proposed for use when estimating plasma volume using the indocyanine green dilution method.

## Background

A method for estimating plasma volume (PV) using indocyanine green (ICG) dilution was first proposed by Bradley [1]. Following ICG injection, ICG binds to plasma proteins and is eliminated almost exclusively by the liver [2]. It takes approximately 2 minutes for ICG concentrations to become well mixed in the plasma, and over the 2- to 5-minute period following injection, the plasma concentrations of ICG can be well approximated by a mono-exponential decay [3]. By fitting the measured plasma ICG concentrations over this time to a mono-exponential decay and using back-extrapolation to obtain the theoretical initial plasma concentration of ICG (ICG_0_, defined as the plasma ICG concentration that would be obtained immediately following injection if there were instant mixing of ICG), the PV can be estimated as:

(1) $$\mathrm{Plasma}\;\mathrm{volume}\;\left(L\right)=\frac{\mathrm{Dose}\;\mathrm{of}\;\mathrm{ICG}\;\mathrm{administered}\;\left(\mathrm{mg}\right)}{\mathrm{Theoretical}\;\mathrm{initial}\;\mathrm{plasma}\;\mathrm{concentration}\;\mathrm{of}\;\mathrm{ICG}\;\left(\mathrm{mg}/L\right)}$$

Different authors have proposed different back-extrapolation methods to estimate ICG_0_ to account for the known properties of ICG removal. In the initial report, Bradley back-extrapolated to time 0 (the ICG injection time) to obtain ICG_0_[1], and this traditional approach has also been used by others [3-6]. Haneda pointed out that the traditional approach of back-extrapolating to t = 0 overestimates ICG_0_ (and therefore underestimates PV), and they proposed back-extrapolating only to t = t_a_, which they defined as the time at which ICG could be measured in the abdominal aorta [2]. Their rationale was that because ICG is excreted primarily by the liver with only minimal extravasation occurring immediately after injection, there should be almost no ICG removal from the circulation prior to t = t_a_. Although measuring the dye concentrations in the abdominal aorta is often not practical experimentally, Haneda suggested that the time it takes ICG to reach the abdominal aorta should be roughly similar to the time it takes to reach peripheral arteries. In principle, frequent samples could be taken from a peripheral artery to estimate t_a_ experimentally; however, this approach does not appear to have been adopted by other researchers.

As an alternative approach to minimize the impact of the time delay associated with ICG transit from the injection site to the heart and the liver, some researchers have used a tourniquet prior to ICG injection to produce reactive hyperemia, thereby enabling more rapid dye mixing in the circulation [5,6].

To better illustrate the potential errors in PV estimation associated with back-extrapolation, Schröder constructed a physical mixing apparatus and performed several experiments to estimate the known system volume [7]. Consistent with the theoretical arguments made by Haneda [2], these experiments demonstrated that back-extrapolating to t = 0 underestimated the system volume and that the error increased with increasing clearance in the system. While the Schröder experiments clearly illustrated the errors in estimating PV associated with back-extrapolation to t = 0, particularly in situations of high clearance, no alternative back-extrapolation method was proposed.

The purpose of this report is to develop an optimal back-extrapolation method for estimating PV using ICG, by developing and utilizing a computational fluid dynamics model of ICG kinetics in human circulation. The computational fluid dynamics model is simulated over a wide range of ICG clearance values to identify a back-extrapolation method that yields accurate estimates of PV over the full range of clearance values.

## Methods

### Mathematical model development

Compartmental models that are commonly used in pharmacokinetic analyses typically assume perfectly mixed compartments and use ordinary differential equations to describe the changes in concentrations within each compartment over time. However, because there is incomplete mixing of ICG in the circulation over the first 2 minutes following ICG injection and the kinetics of ICG during this time are important for estimating ICG_0_, a partial differential equation model describing ICG transit and elimination was developed. This model enables ICG concentrations to vary continuously in both time and space and does not assume perfect mixing within a small number of discrete compartments. The physiologically based mathematical model of ICG kinetics was developed using the following assumptions:

**Figure 1:**
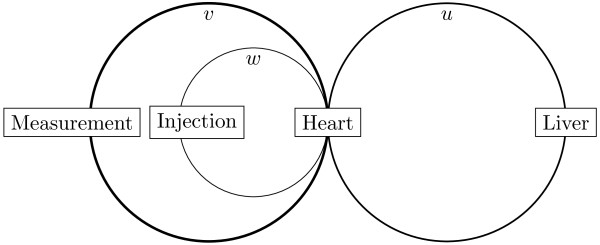
**Schematic view of 3-loop blood flow model.** Blood flow is divided into 3 circulatory loops, with the concentration in each loop varying in both space (length along the loop) and time. The variables *u(t,x)*, *v(t,x)*, and *w(t,x)* represent the concentrations of ICG in Loops 1, 2, and 3, as described in the text. In each loop, the spatial variable (x) runs from 0 to 2π, where x = 0 begins in the center (the heart region), x = π is at the opposite end of the circle (eg, where the liver, injection, and measurement sites are illustrated), and x = 2π is back in the center region. ICG, indocyanine green.

1. ICG is injected into a peripheral vein at t = 0. Prior to t = 0, ICG is not present in the circulation.

2. Injected ICG is transported to the heart by the blood flow in the vein into which it was injected.

3. When blood flow from the vein into which ICG was injected reaches the heart, it is mixed with the blood flow from other vessels and then recirculated to the rest of the body.4. Three different circulatory loops were modeled (Figure 1). Within each loop, the concentration of ICG is calculated at each location and time, and is therefore expressed as a function of both location and time.

a. Loop 1: Hepatic circulation. This represents 30% of the blood flow, and dye is extracted (cleared) from blood flowing through this loop. The concentration of ICG at time *t* and location *x* in this loop is denoted by *u(t,x)* in the equations below.

b. Loops 2 and 3: Nonhepatic circulation. This represents 70% of the blood flow, and there is no clearance of ICG from blood traveling in the nonhepatic circulation. To account for kinetics immediately after injection, the nonhepatic circulation is broken into 2 different loops. There is no clearance of ICG from either of these nonhepatic circulatory loops; the only difference is that ICG is injected directly into 1 of the loops and not into the other.

1) Loop 2: Nonhepatic circulation, excluding the circulatory loop in which the dye is injected. This loop represents 65% of total blood flow, and there is no clearance of ICG from blood flow in this loop. The concentration of ICG in this loop is denoted by *v(t,x)*. Measurements of circulating ICG concentrations are taken at x = π in this loop.

2) Loop 3: Circulation associated with the vein into which ICG is injected. This was modeled to account for 5% of the total blood flow, and there is also no clearance of ICG in this loop. The concentration of ICG in this loop is denoted by *w(t,x)*. The ICG injection occurs at x = π and t = 0.

5. Within each circulatory loop represented, ICG transit occurs through both advection (due to blood flow) and diffusion.

Within each circulatory loop, ICG kinetics are modeled using a 1-dimensional advection diffusion equation; equations of this form are commonly used to model systems in which particles are transferred within a physical system due to both advection and diffusion [8]. The equations used for the 3-loop model shown in Figure 1 are:

(2) $$\frac{\partial u}{\partial t}+c\frac{\partial u}{\partial x}=\nu_{u}\frac{\partial^{2}u}{\partial x^{2}}+\beta \cdot h\left(x\right)\cdot \left(m-u\right)-\alpha \cdot f\left(x\right)\cdot u$$

(3) $$\frac{\partial v}{\partial t}+c\frac{\partial v}{\partial x}=\nu_{v}\frac{\partial^{2}v}{\partial x^{2}}+\beta \cdot h\left(x\right)\cdot \left(m-v\right)$$

(4) $$\frac{\partial w}{\partial t}+c\frac{\partial w}{\partial x}=\nu_{w}\frac{\partial^{2}w}{\partial x^{2}}+\beta \cdot h\left(x\right)\cdot \left(m-w\right);$$

where *m* = *r*_*u*_⋅*u* + *r*_*v*_⋅*v* + *r*_*w*_⋅*w*; *u(t,x), v(t,x),* and *w(t,x)* are the concentrations of ICG at time *t* and location *x* in each of the 3 loops; *c* represents the transit rate for blood through each loop; the ν terms are diffusion coefficients for each loop; *α∙f(x)* represents the clearance of ICG from the hepatic circulatory loop; and the *β∙h(x)* terms represent mixing in the heart region. For each loop, the spatial variable *x* goes from 0 to 2π, and the ICG concentration at x = 0 and x = 2π is the same. The function *f(x)* specifies the region within the hepatic loop where clearance takes place (*f(x)* >0 in a small region meant to represent the liver and *f(x)* = 0 elsewhere). The parameter *α* is used to vary the magnitude of the hepatic clearance of ICG. Similarly, *h(x)* >0 in a small region surrounding x = 0 and x = 2π, meant to represent the heart, and *h(x)* = 0 elsewhere, and β is a parameter that can be adjusted to vary the degree of mixing in the heart. The values of *r*_*u*_*, r*_*v*_*,* and *r*_*w*_ represent the relative blood flows through each loop (*r*_*u*_ *= 0.3*, *r*_*v*_ *= 0.65, r*_*w*_ *= 0.05*), and *m* represents the mean concentration of ICG in the heart region.

**Figure 2:**
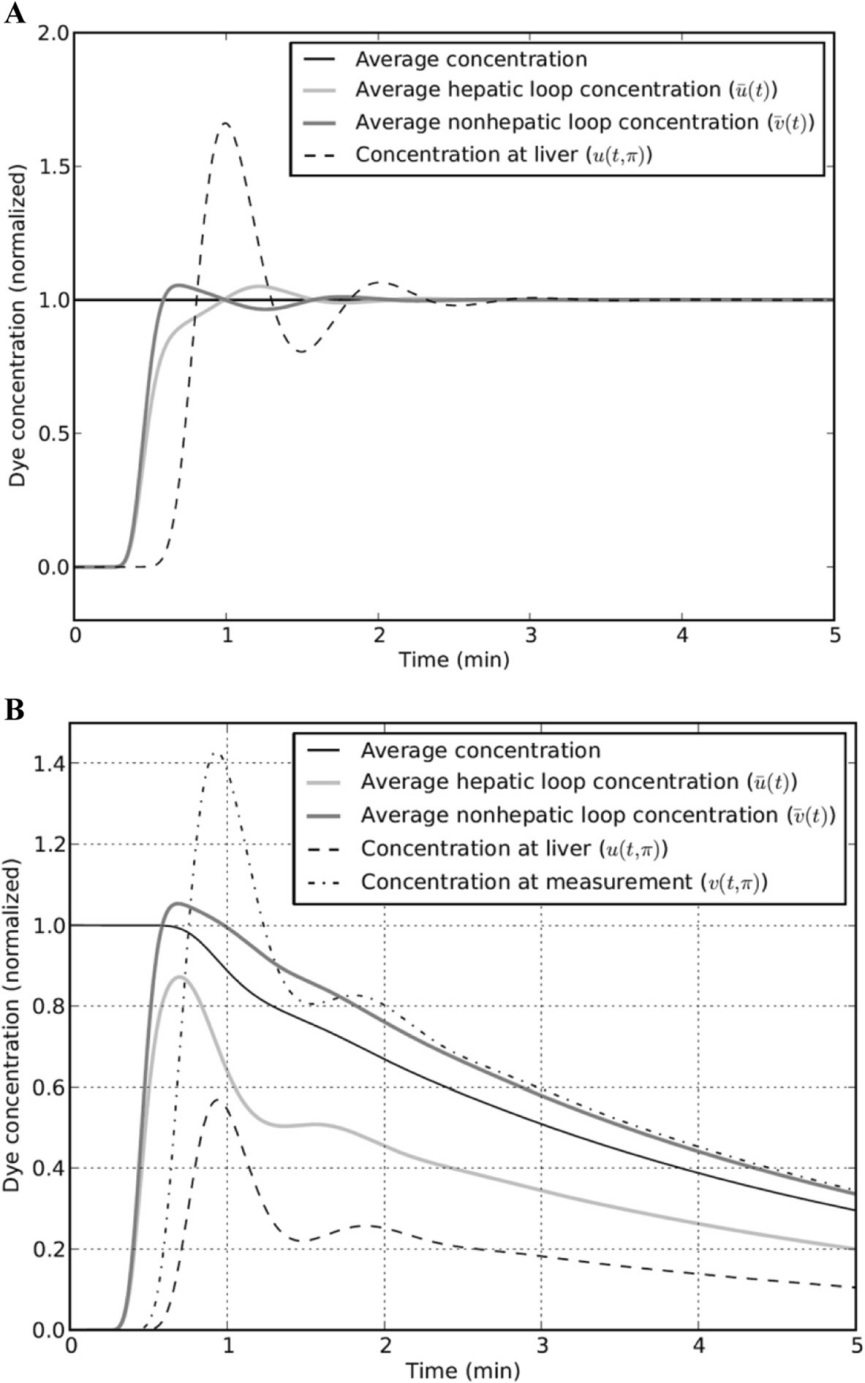
**ICG concentrations for model simulations with no ICG clearance (α = 0) and with hepatic ICG clearance.** Note: For **panel A** (with no ICG clearance), concentrations are normalized so that a value of 1 is equal to the injected dose of ICG divided by the total system volume; for simulations in **panel B** (with hepatic ICG clearance), α = 27. ICG, indocyanine green.

Additional details describing the parameter values and the initial conditions for the model are provided in Additional file 1. In brief, the parameter values for the simulations were chosen to be consistent with the following data:

1. Total blood flow is approximately 5 L/min, and total blood volume is approximately 5 L, so that it takes approximately 1 minute for the blood to circulate through the body [9].

2. Hepatic blood flow is 30% of total blood flow [10].

3. ICG concentrations are well mixed in the plasma by 2 minutes after the injection time [3]. The values of the diffusion coefficients (ν) and the mixing rate in the heart (β) were chosen so that simulated concentrations were well mixed by 2 minutes.

4. The plasma ICG decay rate from 2 to 5 minutes postinjection is consistent with decay rates observed in the clinical study (described below). The parameter associated with hepatic extraction (α) was varied to cover a range of clearance values larger than the range observed in the clinical data.

The partial differential equations were solved using a spectral method as previously described [11]. All calculations were performed in Python, version 2.7. The code used to perform the calculations is included in Additional file 2.

### Clinical study

#### Subjects

Eligible subjects were men and women from 25–70 years of age with type 2 diabetes who were on stable doses of metformin and either an angiotensin-converting enzyme inhibitor (ACEI) or angiotensin receptor blocker (ARB) and who had an estimated glomerular filtration rate ≥70 mL/min/1.73 m^2^. Subjects with conditions or treatments with potential impact on body fluid were excluded from the study (eg, subjects requiring treatment with immunosuppressive therapy; subjects with myocardial infarction, pulmonary hypertension, or New York Heart Association Class III-IV heart failure; subjects with uncontrolled hypertension; subjects taking any antihypertensive medication other than ACEIs or ARBs; and subjects with chronic gastrointestinal disorders). The study was conducted at 1 center in Germany. The protocol and amendment were approved by an Independent Ethics Committee (Ethikkommission der Ärztekammer Nordrhein, Düsseldorf, Germany). All subjects gave written informed consent, in accordance with the Declaration of Helsinki, following institutional guidelines, and in compliance with Good Clinical Practice and regulatory requirements.

#### Materials

ICG was obtained as a powder from PULSION Medical Systems, Munich, Germany, and prepared as a 5 mg/mL solution for injection.

#### Design

This was a randomized, double-blind, placebo-controlled study designed to test the effects of canagliflozin treatment on PV. Only data from the baseline (pretreatment) visit are used in this analysis. Prior to the baseline visit, subjects underwent a 2-week, single-blind, placebo run-in phase and a 3-day diet stabilization period in which subjects received a standardized diet, containing about 200 mEq/day of sodium, while domiciled in the clinical research center. Further details on the study design and subject inclusion and exclusion criteria can be found in reference [12].

#### Procedures

During the baseline visit, subjects were given an intravenous bolus injection of ICG (0.25 mg/kg) over 5 seconds into 1 arm. Blood samples were collected from the other arm every 30 seconds over the period from 2 to 5 minutes after the ICG injection (7 samples) [3].

#### Bioanalytical

Plasma ICG concentrations were determined using a validated high-performance liquid chromatography (HPLC) analytical method at MLM Medical Labs GmbH, Dohrweg 63, 41066 Moenchengladbach, Germany. The inter-assay precision was <7% relative standard deviation, and the accuracy was within ± of nominal concentrations.

#### Model simulations of back-extrapolation algorithms

Several simulations of the model equations were performed to mimic the clinical protocol. In each of the simulations,

1. A specified amount of ICG was injected into Loop 3 at t = 0 into a small region surrounding x = π.

2. The simulated ICG concentrations were “measured” from a peripheral vein every 30 seconds from t = 2 minutes to t = 5 minutes; the (simulated) measurements were taken at x = π in Loop 2.

3. The simulated ICG concentrations from t = 2 to 5 minutes were fitted to a mono-exponential decay by log-transforming the values and using standard linear regression. The relationship was back-extrapolated to t = 0 to obtain the traditional PV estimate.

Normalized concentrations were used in the simulations such that a normalized concentration of 1 is equal to the dose of ICG injected divided by the total system volume.

Similar to the back-extrapolation to t_a_ proposed by Haneda [2], back-extrapolation to alternative times was also performed to account for the time delays associated with the dye traveling to the heart and the liver. The back-extrapolation time that produced the least error between the known volume in the simulations and the volume estimated from the back-extrapolation method over a broad range of clearance values was determined by an iterative search.

#### Calculated PV for clinical study

For each subject, PV was estimated using both the traditional back-extrapolation method (back-extrapolating to t = 0) and the proposed optimal back-extrapolation method. Regression analyses were performed using Matlab, version 8.0.

## Results

### Simulated ICG concentrations with no ICG clearance

To demonstrate some basic properties of the mathematical model, initial simulations were performed with no ICG clearance (α = 0). In this case, the total amount of ICG in the 3 loops remains constant over time. Figure 2A shows the average ICG concentration within each of the circulatory loops at each time point, where the average concentration in a loop is defined as the spatial average along the length of each loop; for example, the average concentration in the hepatic circulation loop is given by:

(5) $$\bar{u}\left(t\right)=\frac{1}{2\pi }\int_{0}^{2\pi }u\left(t,x\right)\mathrm{dx}$$

The curve labeled “average concentration” shows the average ICG concentration in the entire system, which is equal to the total amount of ICG in the 3 loops divided by the sum of the total volume in each loop. As expected, this “average concentration” remains constant over time because there is no clearance. The time delays between ICG injection at t = 0 and the rise in the various concentrations shown in Figure 2A are due to the time delays associated with dye transit from the injection site to the heart and the liver. After a period of approximately 2 minutes, the concentrations are well mixed so that the concentrations at any site in the circulation are approximately equal to the average concentration.

### Simulated ICG concentrations with hepatic ICG clearance

A representative ICG kinetic response when hepatic ICG clearance (as described in Additional file 1) is added to the model is shown in Figure 2B. As in the simulations performed with no clearance, a time delay appears between the ICG injection at t = 0 and the rise in ICG concentrations in the hepatic and nonhepatic circulatory loops. After the initial mixing period, the ICG concentration falls over time in each of the circulatory loops due to ICG clearance in the hepatic circulatory loop. The average concentration for the entire system volume remains at 1 until the injected dye first reaches the liver (at around t = 0.7 minutes) and then falls exponentially after that.

Note that the average ICG concentration in the nonhepatic circulatory loop is always higher than the average ICG concentration in the hepatic circulatory loop due to ICG clearance in the hepatic loop. Therefore, after the initial mixing period, the average ICG concentration in the noncirculatory loop where blood samples are taken is always modestly higher than the average ICG concentration throughout the entire system volume. The difference between the average concentration of ICG in the nonhepatic circulatory loop (ie, $\bar{v}\left(t\right))$ and the overall average concentration increases with increasing amounts of hepatic ICG extraction. This point is discussed further when the sources of error associated with the standard back-extrapolation method are summarized.

**Figure 3:**
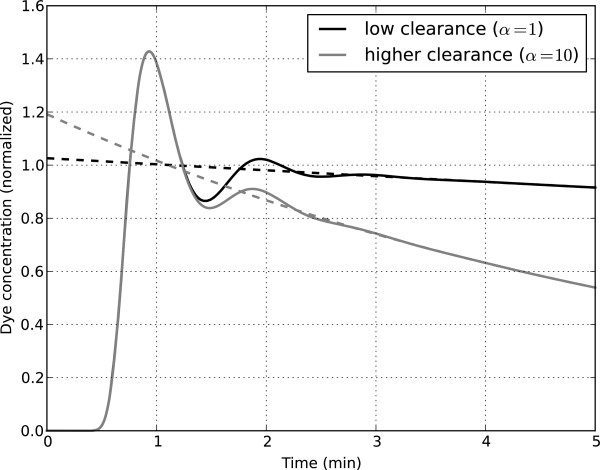
**Illustration of the back-extrapolation method for 2 different clearance rates.** Note: The back-extrapolation for each case was performed by fitting the simulated data from t = 2 to 5 minutes to a mono-exponential decay and then back-extrapolating to t = 0 [3].

Figure 3 illustrates the impact of different clearance rates on the estimated theoretical ICG_0_ values obtained by back-extrapolation and is similar to the results obtained in the physical experiments performed by Schröder [7]. For the 2 different clearance values shown (α = 1 and α = 10), equations 2, 3, 4 and 5 were solved to obtain the simulated measured ICG concentrations (ie, *v(t,π)*) over the period t = 2 to 5 minutes. These “measured” concentrations were then fit to a single exponential decay, as in reference 3, and the exponential functions were back-extrapolated to t = 0 to obtain the estimated ICG_0_ values. In both cases shown, the true value of ICG_0_ = 1. When ICG clearance was low, there was very little error in the estimated ICG_0_ value; but for the higher clearance case, the back-extrapolated ICG_0_ value was approximately 20% higher than the true value of 1. Note that because PV is calculated as Dose/ICG_0_, a 20% overestimation of ICG_0_ will lead to a 17% underestimation of PV.

### Determination of optimal back-extrapolation method

**Figure 4:**
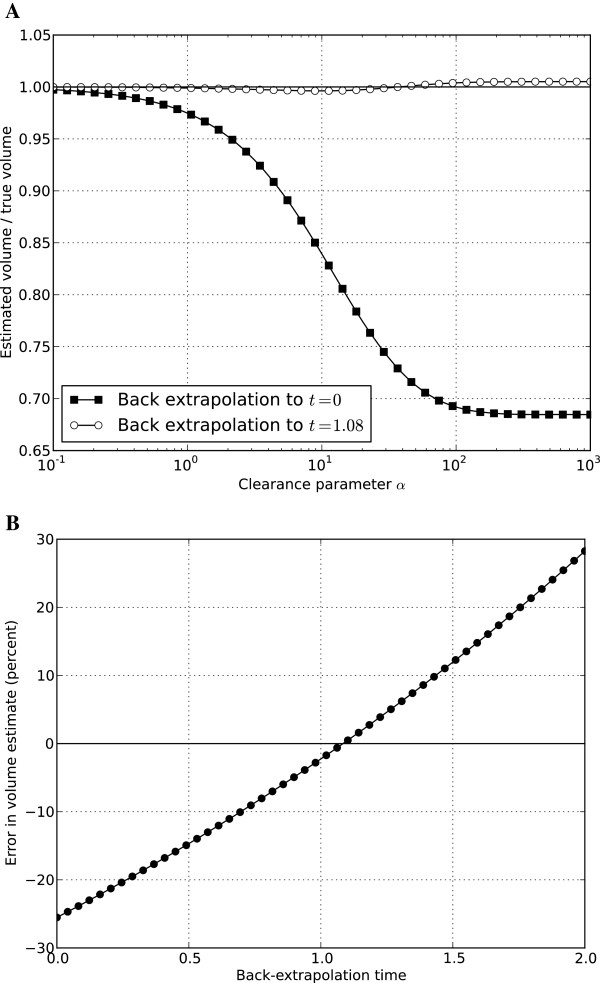
**Errors in estimating plasma volume by back-extrapolation for different clearance rates and different back-extrapolation times.** In **panel A**, the ratio of the estimated PV to the true PV is shown for a range of ICG clearance in the simulations for two different back-extrapolation times (t = 0 and t = 1.08 min). Note that low values of α correspond to low clearance rates and, for values of α >100, there is nearly complete extraction of ICG by the liver. In **panel B**, the percentage error is the estimated PV is shown as a function of the back-extrapolation time used for simulations performed with clearance parameter α = 29 (for this value of α, the simulated plasma ICG profile is similar to the mean plasma ICG profile observed in the clinical study [12]). PV, plasma volume; ICG, indocyanine green.

As shown in Figure 3, back-extrapolation to t = 0 overestimates ICG_0_ (and therefore underestimates PV) when there is hepatic ICG extraction. This is further illustrated in Figure 4A, where the error in the estimated PV is shown as a function of the clearance rate. When back-extrapolating to t = 0, the errors in the estimated PV increase with increasing ICG clearance rates, leading to underestimation of PV by approximately 30% for high hepatic ICG extraction rates. These simulation results are consistent with the experimental results obtained with the physical mixing apparatus developed by Schröder [7].

There are 2 reasons why back-extrapolating to t = 0 overestimates ICG_0_ and therefore underestimates PV:

1. There is a time delay associated with injected dye traveling to the heart and then to the liver. During this period, there is no hepatic ICG extraction, but the back-extrapolation to t = 0 method assumes that the circulating ICG follows the same exponential decay rate beginning at t = 0 as it does when it is well mixed in the circulation.2. As illustrated in Figure 2B, the ICG concentration in the nonhepatic circulatory loop (where blood samples are taken for measuring ICG) is higher than the ICG concentration would be if it was perfectly mixed throughout the entire PV. This is because the ICG concentration in the posthepatic portion of the hepatic circulatory loop is much lower than the ICG concentration in the rest of the circulation.

The magnitude of the error associated with each of these sources of error increases with increasing ICG clearance.

As a result of these errors, alternative back-extrapolation schemes were tested in the simulations. Based on the work of Haneda [2], in which back-extrapolation was done only to the time at which ICG was measured in the abdominal aorta, the following analyses were performed:1. Model simulations of ICG concentrations in the different circulatory loops were performed over the range of clearance values shown in Figure 4A.

2. In each simulation, measurements taken over the period t = 2 to 5 minutes were used to obtain a mono-exponential fit.

3. The exponential fit was back-extrapolated to different time values (denoted as t = t_a_) to estimate the theoretical initial concentration of ICG (assuming perfect mixing) and then calculate the PV (as Dose/ICG(t = t_a_)). The calculated PV obtained by the different back-extrapolation methods was compared to the true system volume.

4. The value of t_a_ that produced the least error over the full range of clearance values was determined by an iterative search.

For the simulations performed, the optimal value of t_a_ was found to be 1.08 minutes. As shown in Figure 4A, by back-extrapolating to t_a_ = 1.08 minutes and using ICG(t = t_a_) when calculating PV, there was virtually no error in the estimated PV over the full range of simulated ICG clearance values.

Note that the optimal value for t_a_ is dependent on several parameters chosen in the simulations, including:

1. The location in the nonhepatic circulatory loop where the dye is injected,

2. The location of the liver in the hepatic circulatory loop,

3. The location where blood samples are taken from the nonhepatic circulatory loop, and

4. The flow rates in the circulatory loops.

In the simulations performed, all of the locations were chosen to be in the middle of the circulatory loops (x = π), and the flow rate in the circulatory loops (circulating once per minute) was consistent with the commonly quoted values of blood flow and blood volume. However, as there is some uncertainty (and likely individual variability) associated with each of these assumptions, a sensitivity analysis was performed to assess how sensitive the estimated PV is to the assumed t_a_ value. This was done by using the simulated results described earlier for a specific case of α = 29 (chosen so that the ICG decay rate in the simulations is similar to decay rates observed in the clinical data) and estimating PV for different back-extrapolation times t_a_. As described earlier, when t_a_ = 0, PV is underestimated by a considerable amount, and when t_a_ = 1.08 minutes, there is virtually no error (Figure 4B). As shown in Figure 4B, as long as t_a_ is between 0.7 and 1.4 minutes, there is less than a 10% error in the estimated PV obtained by the back-extrapolation method. Thus, although the true optimal value of t_a_ will be somewhat uncertain and variable due to the transit times associated with the circulatory flow rates and distances from the injection site to the heart, the liver, and the site of blood samples for measuring ICG, relatively small errors for PV are obtained over a range of t_a_ values.

Based on the simulations performed and the existing uncertainties, it is proposed that a time of t_a_ = 1 minute be used in the back-extrapolation method.

### Clinical study: subject characteristics

Thirty-six subjects were studied. All subjects studied were white, and 31 were male. Demographic and baseline disease characteristics for the subjects are presented in Table 1.

**Table 1:** Demographic and baseline disease characteristics

**Measure**	**Mean (SD)**	**Median (range)**
Age (years)	63 (6)	63 (51–71)
Body weight (kg)	94 (14)	88 (73–130)
Height (cm)	176 (9)	176 (155–192)
BMI (kg/m^2^)	30 (3)	30 (24–37)
HbA_1c_ (%)	7.7 (0.5)	7.7 (7–9)
eGFR (mL/min/1.73 m^2^)	97 (16)	100 (71–132)
Duration of diabetes (years)	9 (4)	8 (2–18)

*SD* standard deviation, *BMI* body mass index, *HbA*_*1c*_ hemoglobin A_1c_, *eGFR* estimated glomerular filtration rate.

### Estimated PV from the clinical study

Individual subject data obtained from the clinical study were used to compare the PV estimates obtained by 2 different back-extrapolation methods. For each subject, the logarithms of the measured ICG values obtained from t = 2 to 5 minutes were calculated, and standard linear regression was used to determine the decay rate. The linear regression relationship provided a good fit to the data in all of the subjects (r^2^ for individual subjects ranged from 0.937–0.998; a figure showing the mean data is provided in Additional file 3: Figure S3). The regression relationship was then used to determine PV by back-extrapolating the regression relationship to either t = 0 or t = 1 minute. The estimated PVs obtained for the 2 different back-extrapolation methods are shown in Table 2.

**Table 2:** Mean (SD) estimates for PV in subjects with type 2 diabetes studied prior to treatment

**Back-extrapolation time**	**N**	**PV (L)**	**PV (mL/kg)**
t = 0	36	2.5 (0.6)	26.8 (5.4)
t = 1 minute	36	3.3 (0.8)	35.1 (7.0)

*SD* standard deviation, *PV* plasma volume, *ICG* indocyanine green.

Note: For each subject, PV was estimated using standard linear regression of the log-transformed ICG concentrations and back-extrapolation to the specified time point for estimation of the theoretical initial concentration of ICG.

The PV estimates obtained when back-extrapolating to t = 0 are below commonly reported PV values of approximately 3 L or approximately 40 mL/kg [13,14], consistent with the results from the simulations and Schröder’s physical experiments showing that back-extrapolation to t = 0 underestimates PV [7]. In contrast, the estimated PVs obtained by back-extrapolating to t = 1 minute are more consistent with commonly reported PV values, particularly when considering that the average body weight of the subjects in the study (94 kg) is higher than the reference 70-kg man for which the 3-L value of PV is commonly quoted.

## Discussion

Several research groups have estimated PV using ICG injections in humans. Following injection, ICG is rapidly bound to plasma proteins and is removed almost exclusively by the liver. Jacob and colleagues utilized a single-compartment model of ICG kinetics over the period from 2 to 5 minutes postinjection to fit the plasma ICG profile using a mono-exponential decay, and then back-extrapolated the exponential function to t = 0 to estimate ICG_0_ and PV [3]. However, several researchers have noted that the traditional approach of back-extrapolating to t = 0 will tend to underestimate PV, largely due to incomplete ICG mixing over the first 2 minutes postinjection and the time lag associated with ICG transit from the injection site to the heart and the liver, where it is extracted [2,7].

Various alternative methods have been proposed to estimate PV, including back-extrapolating only to a time *t*_*a*_ >0, where ICG concentrations could first be measured in the abdominal aorta [2] or using a tourniquet prior to ICG injection to produce reactive hyperemia and speed the mixing of ICG in the circulation [5,6]. In order to better characterize the errors associated with the traditional back-extrapolation method and to develop an improved methodology, a mathematical model of ICG kinetics was developed and simulations were performed to identify a back-extrapolation method that would provide minimal errors in the estimated PV over a wide range of ICG clearance values.

The form of the mathematical model used in the analysis (advection diffusion equations) is commonly used to describe physical systems in which particles are transferred by both convection and diffusion, and is appropriate for modeling ICG kinetics. Simulations were performed to cover a broad range of hepatic ICG clearance rates and, consistent with the physical experiments performed by Schröder [7], the errors in the estimated ICG_0_ values obtained when back-extrapolating to t = 0 increased with increasing clearance rates. This result makes intuitive sense, as back-extrapolating to t = 0 assumes that the same clearance rate exists immediately after injection at t = 0 (when there is no dye at the liver) as when the dye is well mixed in the circulation over the 2- to 5-minute period; the errors associated with this assumption will be higher in cases of high clearance. Based on the work of Haneda [2], alternative back-extrapolation times were tested, and it was demonstrated that if ICG concentrations are back-extrapolated to t = 1 minute rather than to t = 0, only minimal errors in the estimated ICG_0_ (and hence in the estimated PVs) are obtained over a broad range of clearance values.

While the analyses described here provide a practical, model-based method for improving the accuracy of ICG-based estimates of PV, there are some limitations associated with the analyses. The parameter estimates used in the model simulations were based on previously reported mean values rather than being estimated at an individual subject level. In addition, while the simulations covered a broad range of clearance values, the impact of variability in cardiac output and peripheral blood flow distribution [15,16] was not considered in the analyses. Finally, no study was done to directly compare PV estimated using ICG dilution and the proposed back-extrapolation method with PV estimated using a “gold standard” method like ^125^I-albumin [17]. Although these limitations represent areas of potential future work, they are not believed to alter the primary conclusion of this paper that the proposed back-extrapolation scheme is expected to yield more accurate estimates of PV than the traditional back-extrapolation method.

## Conclusions

In summary, based on the analyses performed, the ICG dilution method for estimating PV using the plasma sampling described by Jacob [3] will likely yield more accurate estimates of PV when the theoretical initial ICG concentration is estimated using back-extrapolation to t = 1 minute rather than the traditional approach of back-extrapolating to t = 0.

## Abbreviations

ACEI: angiotensin-converting enzyme inhibitor; ARB: angiotensin receptor blocker; BMI: body mass index; eGFR: estimated glomerular filtration rate; HbA_1c_: hemoglobin A_1c_; HPLC: high-performance liquid chromatography; ICG: indocyanine green; ICG_0_: initial plasma concentration of indocyanine green; PV: plasma volume; SD: standard deviation; T2DM: type 2 diabetes mellitus.

## Competing interests

DP is a full-time employee of Janssen Research & Development, LLC. CR is a part-time consultant for Janssen Research & Development, LLC.

## Authors’ contributions

DP and CR were involved in the modelling design, conducted the analysis, and interpreted the data. Both authors drafted and approved the manuscript.

## Supplementary Material

Additional file 1 **Details of mathematical model used in simulations.** The equations used in the simulations are provided. Click here for file

Additional file 2 **Program files for model simulations.** Text versions of the 2 files used to perform the calculations and generate the figures (titled blood_threeloop.py and specdif.py) are provided. Click here for file

Additional file 3 **Plasma ICG concentrations.** A figure showing mean plasma ICG concentrations for the 36 subjects in the clinical studies is provided. Click here for file
