# Supplementary material for: Optimal back-extrapolation method for estimating plasma volume in humans using the indocyanine green dilution method
Source: Theor Biol Med Model. 2014 Jul 22;11:33. doi: 10.1186/1742-4682-11-33 (PMC4118208; doi:10.1186/1742-4682-11-33)
Supplement: Additional file 1 — Details of mathematical model used in simulations. The equations used in the simulations are provided. [file 1742-4682-11-33-S1.docx]

Additional file 1 – Details of Mathematical Model Used in Simulations

As described in the text, the concentrations of ICG in 3 different circulatory loops are modeled as continuous functions of position along the loop (*x*) and time (*t*). The equations used in the simulations are shown below.

$$\begin{matrix} \frac{\partial u}{\partial t}+c\frac{\partial u}{\partial x}=\nu_{u}\frac{\partial^{2}u}{\partial x^{2}}+\beta\cdot h(x)\cdot(m-u)-\alpha\cdot f(x)\cdot u \\ \frac{\partial v}{\partial t}+c\frac{\partial v}{\partial x}=\nu_{v}\frac{\partial^{2}v}{\partial x^{2}}+\beta\cdot h(x)\cdot(m-v) \\ \frac{\partial w}{\partial t}+c\frac{\partial w}{\partial x}=\nu_{w}\frac{\partial^{2}w}{\partial x^{2}}+\beta\cdot h(x)\cdot(m-w) \\ \text{where }m=r_{u}\cdot u+r_{v}\cdot v+r_{w}\cdot w \end{matrix}$$

on a periodic domain 0 ≤ *x* ≤ 2π, where

*u, v,* and *w* are the concentrations of ICG in the 3 circulatory loops shown in Figure 1

ν_u_, ν_v_, and ν_w_ are the diffusion coefficients for each loop

*c* is the convection speed associated with the blood flow in each loop

*r_u_*, *r_v_*, and *r_w_* are the relative amounts of blood flow in each loop (*r_u_* + *r_v_* + *r_w_* = 1)

*β* is a parameter used to determine the rate of mixing in the heart region

*α* is a parameter used to determine the rate of hepatic extraction of ICG

*h(x)* and *f(x)* are functions used to specify the regions in the loops where mixing and extraction occur

A value of *c*=2π was used in all simulations. With this value of *c* (and the length of each loop equal to 2π), it takes particles 1 minute to transit through each loop, consistent with the values associated with human blood flow (total blood volume and cardiac output of approximately 5 L and 5 L/min, respectively). The values of *r_u_*, *r_v_*, and *r_w_* were 0.3, 0.65, and 0.05 as described in the text.

The diffusion coefficients ν_u_, ν_v_, and ν_w_ were set to *r_u_*ν, *r_v_*ν, and *r_w_*ν, respectively, with ν=5, and the value of *β* was 800. These values were selected so that circulating ICG concentrations would be generally well mixed by t=2 minutes (see Figure 2A).

The functions *h(x)* and *f(x)* were modeled as smoothed versions of step functions that centered on the regions of *x=­*0 and *x­*=π, respectively (Supplemental Figure S1). The equations used are shown below.

$$h(x)=\left\{\begin{matrix} 0.5\times\left(1+\tanh(x/dx)\right)\text{ if -w}_{h}\text{/2 }\leq\text{x }\leq\text{ w}_{h}\text{/2} \\ 0\text{ otherwise} \end{matrix}\right.\text{ }$$

$$f(x)=\left\{\begin{matrix} 0.5\times\left(1+\tanh((x-\pi)/dx)\right)\text{ if }\pi\text{-w}_{f}\text{/2 }\leq\text{x }\leq\text{ }\pi+\text{w}_{f}\text{/2} \\ 0\text{ otherwise} \end{matrix}\right.\text{ }$$

where *w_h_* and *w_f_* are parameters describing the width of the heart and liver regions in the model and *dx* is the grid spacing used in the simulations. The value of *w_h_* was set to 300 mL/5000 mL ∙2π ≈ 0.377 based on the approximate volume of the blood in the heart (300 mL) divided by the total blood volume (5000 mL) and the total distance associated with the circulatory loops (2π). The value of *w_f­_* was set to 0.5. A grid size of N=200 points was used for each circulatory loop and the value of *dx _­_*was *dx =* 2π/N.

Supplemental Figure S1. Graphical representation of functions *h(x)* and *f(x)* used to describe the regions in the circulatory loops where mixing (heart region) and extraction (liver region) of dye occur.

The equations were solved using a spectral method in which the derivatives were defined using Fourier interpolants as in Weideman 2000, converting the set of partial differential equations into a set of linear ordinary differential equations. The corresponding system of ordinary differential equations was then solved exactly for discrete time points to obtain the concentrations of ICG at each of the discrete space and time points. The initial conditions used in the simulations to represent a bolus injection of dye at t=0 in the region near w=π are shown in Supplemental Figure S2; the value of *w(0,x)* was set to a Gaussian centered at x= π with a standard deviation of 0.05.

Supplemental Figure S2. Initial conditions for ICG concentrations in the circulatory loop in which ICG is injected.

ICG, indocyanine green.
