# Supplementary material for: Optimal back-extrapolation method for estimating plasma volume in humans using the indocyanine green dilution method
Source: Theor Biol Med Model. 2014 Jul 22;11:33. doi: 10.1186/1742-4682-11-33 (PMC4118208; doi:10.1186/1742-4682-11-33)
Supplement: Additional file 2 — Program files for model simulations. Text versions of the 2 files used to perform the calculations and generate the figures (titled blood_threeloop.py and specdif.py) are provided. [file 1742-4682-11-33-S2.docx]

Additional file 2 – Program Files for Model Simulations

Two files were used to perform the calculations and generate the figures (titled blood_threeloop.py and specdif.py). Text versions of these files are provided below.

File: blood_threeloop.py

#!/usr/bin/env python

#

# blood_threeloop.py - run 3-loop PDE model for dye clearance in blood

# Clancy Rowley, June 2012

import numpy as np

from scipy import linalg

from specdif import fourdif

import matplotlib.pyplot as plt

class BloodModel(object):

def integrate(self, dt, T):

Ad = linalg.expm(dt * self.A)

C = self.C

t = np.arange(0., T+dt/2.0, dt)

nt = len(t)

nout = C.shape[0]

y = np.zeros((nt,nout))

q = self.x0

y[0,:] = np.dot(C, q)

for i in xrange(1,nt):

q = np.dot(Ad, q)

y[i,:] = np.dot(C, q)

return t,y

def normal(self, x0, sig):

"""Return normal distribution centered at x0, with stdev sig"""

n = 3 # number of shifted copies to add

x = self.x

u = np.zeros(len(x))

twopi = 2*np.pi

for j in xrange(-n,n+1):

u = u + np.exp(-(x - x0 - twopi * j)**2 / sig**2 / 2.0)

u = u / np.sqrt(twopi * sig**2)

return u

def smooth_pulse(self, a, b):

"""return a function that is 1 between a and b (mod 2pi),

and zero elsewhere, smoothly interpolating at boundaries.

Integral of this function from 0 to 2pi should be (b-a)"""

x = self.x

dx = x[1] - x[0]

y = np.zeros(len(x))

twopi = 2 * np.pi

a = a % twopi

b = b % twopi

def step(c):

return (1 + np.tanh((x - c) / dx)) / 2.0

if b > a:

# 0 for x < a, 1 for a < x < b, 0 for x > b

return step(a) * (1 - step(b))

else:

# 1 for x < b, 0 for b < x < a, 1 for x > a

return 1 - step(b) * (1 - step(a))

def calculate_eigenvalues(self):

# lam = np.linalg.eigvals(self.A)

lamv, V = np.linalg.eig(self.A)

V = np.array(V) # convert from matrix to array

indv = np.argmax(np.real(lamv))

self.lam_max = float(np.real(lamv[indv]))

lamw, W = np.linalg.eig(self.A.transpose())

W = np.array(W)

indw = np.argmax(np.real(lamw))

v1 = np.real(V[:,indv])

w1 = np.real(W[:,indw])

c = np.dot(self.C, v1.transpose()) / np.dot(w1, v1)

self.k_extrap = np.outer(c, w1)

self.v_max = v1

def extrapolate_ic(self, x0):

"""Return initial concentration predicted from back

extrapolation, for initial condition x0"""

return np.dot(self.k_extrap, x0)

def back_extrapolate(self, t, t0):

"""Return back-extrapolated concentration

t is a numpy array of times

t0 is the time to stop back-extrapolating"""

a0 = self.extrapolate_ic(self.x0)

y = np.outer(np.exp(self.lam_max * t), a0)

j = np.where(t > t0)[0][0]

y[:j,:] = y[j,:]

return y

def clearance(self):

"""Return clearance, as a fraction of total flow rate

Clearance is defined as V * lam, where V is fluid volume and

lam is exponential decay rate.

Total flow rate = V / T where T is flow-through time

clearance = lam * T"""

T_period = 2 * np.pi / self.c

return -self.lam_max * T_period

class ThreeLoopModel(BloodModel):

def __init__(self, N, c, nu, alpha, beta, w1, w2, vols,

x_meas = 3. * np.pi / 2.0):

"""

N: number of gridpoints

c: convection speed

nu: diffusion coefficient

(diffn. coef.) in each loop is volume * nu

alpha: clearance rate

beta: mixing rate

w1: width of clearance region

w2: width of mixing region

vols: tuple of volumes of (injection, clearance) loops

x_meas: measurement location in non-clearance loop (between 0 and 2pi)

outputs from C matrix are:

overall concentration of dye

concentration of dye in loop 1 (hepatic loop, with clearance)

concentration of dye in loop 2 (nonhepatic loop, no clearance)

concentration of dye in loop 3 (injection)

concentration at liver

concentration of dye at measurement location (in loop 2)

"""

x, D = fourdif(N, 1)

x, D2 = fourdif(N, 2)

self.x = x

f = self.smooth_pulse(np.pi - w1/2., np.pi + w1/2.)

# mixing region

h = self.smooth_pulse(-w2/2., w2/2.)

betaH = np.diag(beta * h)

rw, ru = vols

rv = 1 - (ru + rw)

Auu = -c * D + ru * nu * D2 - alpha * np.diag(f) - (1-ru) * betaH

Auv = rv * betaH

Auw = rw * betaH

Avu = ru * betaH

Avv = -c * D + rv * nu * D2 - (1-rv) * betaH

Avw = Auw

Awu = Avu

Awv = Auv

Aww = -c * D + rw * nu * D2 - (1-rw) * betaH

self.A = np.bmat([[Auu, Auv, Auw],

[Avu, Avv, Avw],

[Awu, Awv, Aww]])

conc_u = np.r_[np.ones(N) / float(N), np.zeros(2*N)]

conc_v = np.r_[np.zeros(N), np.ones(N) / float(N), np.zeros(N)]

conc_w = np.r_[np.zeros(2*N), np.ones(N) / float(N)]

conc = ru * conc_u + rv * conc_v + rw * conc_w

# point measurement at x=pi in liver loop

conc_f = np.zeros(3*N)

ind = np.where(x > np.pi)[0][0]

conc_f[ind] = 1

# point measurement at x = 3pi/2

conc_y = np.zeros(3*N)

ind = np.where(x > x_meas)[0][0]

conc_y[N + ind] = 1

self.C = np.array([conc, conc_u, conc_v, conc_w, conc_f, conc_y])

self.x0 = np.zeros(3*N)

self.x0[2*N:] = 2 * np.pi * self.normal(np.pi, 0.05) / rw

self.f = f

self.h = h

self.c = c

self.r = (ru, rv, rw)

self.calculate_eigenvalues()

def back_extrapolate_old(t, y, lam):

t1 = 2 # time to begin back-extrapolation

j = np.where(t > t1)[0][0]

c = y[j]

return np.exp(lam * (t - t1)) * c

def back_extrapolate_new2(t, y, lam):

t1 = 2 # time to begin back-extrapolation

t0 = 0.5 # delay time

j = np.where(t > t1)[0][0]

c = y[j]

y_extrap = np.exp(lam * (t - t1)) * c

k = np.where(t > t0)[0][0]

y_extrap[:k] = y_extrap[k]

return y_extrap

##

## Define parameters for 3-loop model

##

N = 200 # number of gridpoints in each loop

c = 2 * np.pi # convection speed

nu = 5. # diffusion coefficient

vols = (0.05, 0.3) # volume ratios: (injection, clearance) to total blood flow

alpha = 29. # rate at which liver clears dye

beta = 800. # rate at which heart mixes dye

w1 = 0.5 # width of clearance region (liver)

w2 = 0.377 # width of mixing region (heart: 300 mL / 5L * 2pi)

x_meas = np.pi

model = blood.ThreeLoopModel(N, c, nu, alpha, beta, w1, w2, vols, x_meas)

def non_clearance_style(p):

p.set_color("0.5")

p.set_linewidth(2)

def clearance_style(p):

p.set_color("0.75")

p.set_linewidth(2)

def plot_liver_region():

plot_oneloop(model.f, 'Liver amplitude $f(x)$', 'liver.eps',

ylim=[0,1.1])

def plot_heart_region():

plot_oneloop(model.h, 'Heart amplitude $h(x)$', "heart.eps",

ylim=[0,1.1])

def plot_ic():

w0 = model.x0[2*N:]

plot_oneloop(w0, 'Initial concentration $w(0,x)$', "ic.eps")

def no_clearance():

dt = 0.01

T = 5

t,y = model_noliver.integrate(dt,T)

plt.clf()

p, = plt.plot(t, y[:,0], "k-")

p, = plt.plot(t, y[:,1])

clearance_style(p)

p, = plt.plot(t, y[:,2])

non_clearance_style(p)

plt.plot(t, y[:,4], "k--")

plt.ylim(-0.2,2.0)

plt.xlabel('Time (min)')

plt.ylabel('Dye concentration (normalized)')

plt.legend(('Average concentration',

r'Average hepatic loop concentration ($\bar u(t)$)',

r'Average nonhepatic loop concentration ($\bar v(t)$)',

r'Concentration at liver ($u(t,\pi)$)'), prop={'size':12})

plt.savefig("three_loop_no_clearance.eps")

return t,y

def plot_concentration():

dt = 0.01

T = 5

t,y = model.integrate(dt,T)

plt.clf()

p, = plt.plot(t, y[:,0], 'k-')

p, = plt.plot(t, y[:,1])

clearance_style(p)

p, = plt.plot(t, y[:,2])

non_clearance_style(p)

p, = plt.plot(t, y[:,4],"k--")

p, = plt.plot(t, y[:,5], "k-.")

# timestep of max measured concentration

jmax = np.argmax(y[:,5])

tmax = t[jmax]

print "Max measured concentration at t = %f" % tmax

plt.ylim(0,1.5)

plt.grid(True)

plt.xlabel('Time (min)')

plt.ylabel('Dye concentration (normalized)')

plt.legend(('Average concentration',

r'Average hepatic loop concentration ($\bar u(t)$)',

r'Average nonhepatic loop concentration ($\bar v(t)$)',

'Concentration at liver ($u(t,\pi)$)',

'Concentration at measurement ($v(t,\pi)$)'))

plt.savefig("three_loop.eps")

def plot_measured_concentration(alpha_list):

clearance = []

lines = []

plt.clf()

color_list = ["0.0","0.5"]

lw = 2

for alpha, color in zip(alpha_list, color_list):

model = blood.ThreeLoopModel(N, c, nu, alpha, beta, w1, w2,

vols, x_meas)

dt = 0.01

T = 5

t,y = model.integrate(dt,T)

l, = plt.plot(t, y[:,5])

l.set_color(color)

l.set_linewidth(lw)

lines.append(l)

clearance.append(model.clearance())

y_extrap = model.back_extrapolate(t, 0)

l, = plt.plot(t, y_extrap[:,5],'--')

l.set_color(color)

l.set_linewidth(lw)

plt.ylim(0,1.6)

plt.grid(True)

plt.xlabel('Time (min)')

plt.ylabel('Dye concentration (normalized)')

plt.legend(lines, [r"low clearance ($\alpha = %.0f$)" % alpha_list[0],

r"higher clearance ($\alpha = %.0f$)" % alpha_list[1]])

plt.savefig("three_loop_compare.eps")

def plot_extrap_errors(alpha_vals):

plot_vs_clearance = False # if True, plot vs. clearance

# if False, plot vs. alpha

t1 = 1.08 # time to extrapolate back to, for new method

T_period = 1 # flow-through time

percent = 100

clearance = np.zeros(len(alpha_vals))

vol_old = np.zeros(len(alpha_vals))

vol_new = np.zeros(len(alpha_vals))

for j, alpha in enumerate(alpha_vals):

print "alpha = %.2f" % alpha

model = blood.ThreeLoopModel(N, c, nu, alpha, beta, w1, w2, vols, x_meas)

clearance[j] = -model.lam_max * T_period * percent

k = model.extrapolate_ic(model.x0)

# k[0] is extrapolated average concentration

# k[5] is extrapolated measured concentration

vol_old[j] = 1. / k[5]

vol_new[j] = 1. / (k[5] * np.exp(model.lam_max * t1))

plt.clf()

if plot_vs_clearance:

# plot vs. clearance as % of system flow

plt.plot(clearance, vol_old, 'k-s')

plt.plot(clearance, vol_new, 'k-o', markerfacecolor="w")

plt.xlabel('Clearance (% of system flow)')

else:

plt.semilogx(alpha_vals, vol_old, 'k-s')

plt.semilogx(alpha_vals, vol_new, 'k-o', markerfacecolor="w")

plt.xlabel(r'Clearance parameter $\alpha$')

plt.axhline(y=1, color='k')

plt.grid(True)

plt.ylabel('Estimated volume / true volume')

plt.legend(['Back extrapolation to $t=0$',

'Back extrapolation to $t=%.2f$' % t1], 'lower left')

plt.savefig('extrap_errors.eps')

def plot_volume_err_vs_extrap_time(extrap_times):

percent = 100

print "clearance: %.3f%%" % (model.clearance() * percent)

k = model.extrapolate_ic(model.x0)

c0 = k[5]

lam = model.lam_max

times = np.array(extrap_times)

errors = np.array([1. / (c0 * np.exp(lam * t)) - 1.0 for t in times])

plt.clf()

plt.plot(times, errors * percent,'k-o')

plt.grid(True)

plt.axhline(y=0, color='k')

plt.xlabel("Back-extrapolation time")

plt.ylabel("Error in volume estimate (percent)")

plt.savefig("errors_vs_times.eps")

if __name__ == "__main__":

print("Executing script for 3-loop model")

plot_liver_region()

plot_heart_region()

plot_ic()

no_clearance()

plot_concentration()

plot_measured_concentration((1,10))

alpha_list = np.logspace(-1,3,40)

plot_extrap_errors(alpha_list)

extrap_times = np.linspace(0,2,50)

plot_volume_err_vs_extrap_time(extrap_times)

File: specdif.py

import numpy as np

from scipy import linalg

def fourdif(N, m):

"""

The function [x, DM] = fourdif(N,m) computes the m'th derivative Fourier

spectral differentiation matrix on grid with N equispaced points in [0,2pi)

Input:

N: Size of differentiation matrix.

m: Order of derivative required (non-negative integer)

Output:

x: Equispaced points 0, 2pi/N, 4pi/N, ... , (N-1)2pi/N

DM: m'th order differentiation matrix

Explicit formulas are used to compute the matrices for m=1 and 2.

A discrete Fouier approach is employed for m>2. The program

computes the first column and first row and then uses the

toeplitz command to create the matrix.

For m=1 and 2 the code implements a "flipping trick" to

improve accuracy suggested by W. Don and A. Solomonoff in

SIAM J. Sci. Comp. Vol. 6, pp. 1253--1268 (1994).

The flipping trick is necessary since sin t can be computed to high

relative precision when t is small whereas sin (pi-t) cannot.

S.C. Reddy, J.A.C. Weideman 1998. Corrected for MATLAB R13

by JACW, April 2003.

Translated to Python by Clancy Rowley, 28 May 2012

"""

h = 2 * np.pi / N # grid spacing

x = h * np.arange(N) # gridpoints

kk = np.arange(1,N)

n1 = np.floor((N-1)/2.0)

n2 = np.ceil((N-1)/2.0)

if m == 0: # compute first column

col1 = np.zeros(N) # of zeroth derivative

col1[0] = 1 # matrix, which is identity

row1 = col1

elif m == 1: # compute first column

if N % 2 == 0: # of 1st derivative matrix

topc = 1./np.tan(np.arange(1,n2+1) * h/2.0)

col1 = np.zeros(N)

col1[1:] = 0.5 * (-1)**kk * np.concatenate((topc, -np.flipud(topc[:n1])))

else:

topc = 1./np.sin(np.arange(1,n2+1) * h/2.0)

col1 = np.zeros(N)

col1[1:] = 0.5 * (-1)**kk * np.concatenate((topc, np.flipud(topc[:n1])))

row1 = -col1; # first row

elif m == 2: # compute first column

if N % 2 == 0: # of 2nd derivative matrix

topc = 1. / np.sin(np.arange(1,n2+1) * h/2.0)**2

col1 = np.zeros(N)

col1[0] = -np.pi**2 / 3. / h**2 - 1./6.

col1[1:] = -0.5 * (-1)**kk * np.concatenate((topc, np.flipud(topc[:n1])))

else:

topc = 1. / (np.sin(np.arange(1,n2+1) * h/2.0) *

np.tan(np.arange(1,n2+1) * h/2.0))

col1 = np.zeros(N)

col1[0] = -np.pi**2 / 3.0 / h**2 + 1./12.

col1[1:] = -0.5 * (-1)**kk * np.concatenate((topc, -np.flipud(topc[:n1])))

row1=col1; # first row

else:

raise NotImplementedError("derivs higher than 2 not implemented")

# below is Matlab code for computing higher derivatives using FFT

"""

else % employ FFT to compute

N1=floor((N-1)/2); % 1st column of matrix for m>2

N2 = (-N/2)*rem(m+1,2)*ones(rem(N+1,2));

mwave=1j*[(0:N1) N2 (-N1:-1)];

col1=real(ifft((mwave.^m).*fft([1 zeros(1,N-1)])));

if rem(m,2)==0,

row1=col1; % first row even derivative

else

col1=[0 col1(2:N)]';

row1=-col1; % first row odd derivative

end;

end;

"""

DM = linalg.toeplitz(col1, row1)

return x, DM
