# Supplementary material for: Optimal back-extrapolation method for estimating plasma volume in humans using the indocyanine green dilution method
Source: Theor Biol Med Model. 2014 Jul 22;11:33. doi: 10.1186/1742-4682-11-33 (PMC4118208; doi:10.1186/1742-4682-11-33)
Supplement: Additional file 3 — Plasma ICG concentrations. A figure showing mean plasma ICG concentrations for the 36 subjects in the clinical studies is provided. [file 1742-4682-11-33-S3.docx]

**Additional file 3 – Plasma ICG Concentrations**

Figure S3. Mean (±SD) of the natural logarithm of plasma ICG concentrations for the 36 subjects in the clinical study. Plasma ICG concentrations were measured in µg/ml.

ICG, indocyanine green.
